# Developmental up-regulation of NMDA receptors in the prefrontal cortex and hippocampus of mGlu5 receptor knock-out mice

**DOI:** 10.1186/s13041-021-00784-9

**Published:** 2021-05-07

**Authors:** Tiziana Imbriglio, Remy Verhaeghe, Nico Antenucci, Stefania Maccari, Giuseppe Battaglia, Ferdinando Nicoletti, Milena Cannella

**Affiliations:** 1 IRCCS Neuromed, Pozzilli, IS Italy; 2 Department of Physiology and Pharmacology “V. Erspamer”, University Sapienza of Rome, Piazzale Aldo Moro, 5, 00185 Rome, Italy; 3 CNRS, UMR 8576, UGSF, Unité de Glycobiologie Structurale et Fonctionnelle, University of Lille, Lille, France

**Keywords:** Interneuron related genes, NMDA receptor subunits, Prefrontal cortex, Hippocampus MK-801, Locomotor activity

## Abstract

mGlu5 metabotropic glutamate receptors are highly expressed and functional in the early postnatal life, and are known to positively modulate NMDA receptor function. Here, we examined the expression of NMDA receptor subunits and interneuron-related genes in the prefrontal cortex and hippocampus of mGlu5^−/−^ mice and wild-type littermates at three developmental time points (PND9, − 21, and − 75). We were surprised to find that expression of all NMDA receptor subunits was greatly enhanced in mGlu5^−/−^ mice at PND21. In contrast, at PND9, expression of the GluN2B subunit was enhanced, whereas expression of GluN2A and GluN2D subunits was reduced in both regions. These modifications were transient and disappeared in the adult life (PND75). Changes in the transcripts of interneuron-related genes (encoding parvalbumin, somatostatin, vasoactive intestinal peptide, reelin, and the two isoforms of glutamate decarboxylase) were also observed in mGlu5^−/−^ mice across postnatal development. For example, the transcript encoding parvalbumin was up-regulated in the prefrontal cortex of mGlu5^−/−^ mice at PND9 and PND21, whereas it was significantly reduced at PND75. These findings suggest that in mGlu5^−/−^ mice a transient overexpression of NMDA receptor subunits may compensate for the lack of the NMDA receptor partner, mGlu5. Interestingly, in mGlu5^−/−^ mice the behavioral response to the NMDA channel blocker, MK-801, was significantly increased at PND21, and largely reduced at PND75. The impact of adaptive changes in the expression of NMDA receptor subunits should be taken into account when mGlu5^−/−^ mice are used for developmental studies.

## Introduction

One of the earliest discovery in the field of metabotropic glutamate (mGlu) receptors was that glutamate-stimulated polyphosphoinositide (PI) hydrolysis was prominent in the first 9–10 days of postnatal life, and then progressively declined to become negligible in the adult life [1]. Subsequent studies have shown that the large PI response to glutamate early after birth was mediated by the mGlu5 receptor, one of the two receptor subtypes coupled to G_q/11_ [2, 3]. mGlu5 receptors are highly expressed in the early postnatal life in most of the forebrain regions, and expression declines afterwards [4, 5] with a developmental shift from mGlu5a to mGlu5b splice variants in the cerebral cortex, hippocampus and corpus striatum [6]. This evidence suggests that mGlu5 receptors are involved in mechanisms that shape the developmental trajectory of the CNS in the postnatal life. Of note, mGlu5 receptors are drug candidate targets for the treatment of neurodevelopmental disorders, such as monogenic autism and schizophrenia [7–15]. mGlu5 receptors are expressed on cortical and hippocampal GABAergic interneurons, which coordinate network oscillations and feedback/feedforward inhibition mechanisms [16, 17]. Genetic deletion of mGlu5 receptors causes alterations in the expression of interneuron-related genes in the prefrontal/frontal cortex and hippocampus of adult mice [18], and disrupted latent inhibition [19]. In addition, postnatal ablation of mGlu5 receptors from parvalbumin (PV)-positive interneurons caused a schizophrenia-like phenotype in adult mice, characterized by impaired rhythmic cortical oscillatory activity and alterations in sensory-motor gating, learning and memory, and social recognition [20].

Little is known on how the absence of mGlu5 receptors affects the biology of interneurons across postnatal development. We have found recently that the density of perineuronal nets (PNNs), which are specialized formations of the extracellular matrix surrounding parvalbumin-positive (PV^+^) interneurons, was largely increased in the somatosensory cortex of mice lacking mGlu5 receptors at PND16 [21], suggesting a role for mGlu5 receptors in mechanisms of developmental plasticity of cortical interneurons.

Here, we examined the expression of biochemical makers of GABAergic interneurons in the prefrontal cortex and hippocampus of mGlu5 receptor knockout mice at three time points of postnatal development (PND9, PND21, and PND75). In addition we measured the expression of the NMDA receptor subunits for the following reasons: (1) NMDA receptors are largely expressed in cortical and hippocampal interneurons, are constitutively active in fast-spiking PV^+^ interneurons, and are critically involved in the generation of gamma frequency oscillations and behavior [22–25]; and, (2) NMDA receptors are physically and functionally linked to mGlu5 receptors [26–32], and the cross-talk between the two receptors is regulated by the dynamics of the mGlu5/Homer protein complex in dendritic spines [33]. We report that all NMDA receptor subunits are overexpressed in the prefrontal cortex and hippocampus of mGlu5 receptor knockout mice at PND21, but not in the adult life. Developmental alterations in the expression of NMDA receptor subunits were paralleled by changes in MK-801-induced locomotor hyperactivity, which reflects the inhibition of NMDA receptors expressed by GABAergic interneurons.

## Material and methods

### Animals

mGlu5 receptor knockout B6;129-Grm5^tm1Rod^/J (mGluR5^−/−^) mice [34] were purchased from The Jackson Laboratory (Bar Harbor, ME, USA). Wild-type and knockout mice were generated by mating male and female heterozygous parents, with the genotypes determined by PCR (Jackson lab protocol). We used mGlu5^−/−^ mice and wild-type littermates of both sex at PND9, and exclusively male mice at PND21 and PND75. Mice were housed in an animal care facility at 23 °C on a 12 h light/12 h dark cycle with food and water provided ad libitum. All mice that we used were sacrificed by cervical dislocation. Experiments were performed following the Guidelines for Animal Care and Use of the National Institutes of Health to minimize the number of animals and animal suffering. The experimental protocol was approved by the Ethical Committee of Neuromed Institute (Pozzilli, Italy) and by the Italian Ministry of Health. Animals at PND9, PND21, and PND75 were used for biochemical analysis of interneuron-related genes and NMDA receptor subunits. Animals at PND21, and PND75 were used for the assessment of MK-801 induce locomotor activity.

### Measurements of mRNA levels of interneuron-related genes

Mice were killed by decapitation at PND9, PND21 or PND75. The brains were removed and the prefrontal cortex and hippocampus immediately dissected and frozen on liquid nitrogen. Total RNA was extracted using the Trizol reagent (Invitrogen, Carlsbad, CA) according to manufacturer’s instructions. The RNA was further treated with DNase (Qiagen, Hilden, Germany), and single strand cDNA was synthesized from 1.5 μg of total RNA using Superscript III (Invitrogen) and random hexamers as previously described [35]. Real-time PCR was performed on 15 ng of cDNA by using specific primers and Power SYBR Green Master Mix (Biorad, Hercules, CA) on an Applied Biosystems Step-One instrument.

Thermal cycler conditions were as follows: 10 min at 95 °C, 40 cycles of denaturation (15 s at 95 °C), and combined annealing/extension (1 min at 58–60 °C). The sequence of the specific primers is shown in Table 1: mRNA copy number for each gene was calculated from serially diluted standard curves simultaneously amplified with the samples and normalized with respect to the transferrin receptor (TFRC) mRNA copy number. Each sample was analyzed in duplicate together with two negative controls.

**Table 1:** Primer sequences used for real-time PCR analysis

Name	Primer	Seq 5′– > 3′
Gad1 (GAD67)	Forw	GTACTCCTGTGACAGAGCCG
	Rev	GTATTAGGATCCGCTCCCGC
Gad2 (GAD65)	Forw	GAGCTGCAGCCTTAGGGATT
	Rev	GCACTCACCAGGAAAGGAAC
Grin1 (GluN1)	Forw	AACCTGCAGCAGTACCATCC
	Rev	GCAGCAGGACTCATCAGTGT
Grin2A(GluN2A)	Forw	TCTCCGCCTTTCCGATTTGG
	Rev	TGGCAAAGATGTACCCGCTC
Grin2B (GluN2B)	Forw	CGCTCTCCACACCCTGAGAT
	Rev	TAGAAGCCAAAGCTCTAGGC
Grin2C (GluN2C)	Forw	CATTAGGGATTTCCCCAAACGC
	Rev	ACCTTCCTAGTCCAAGCACA
Grin2D (GluN2D)	Forw	TCCTGGGGGACGATGAGATT
	Rev	AGTCGCCAGTACACAAGGTG
Pvalb (parvalbumin)	Forw	GCTTCTCCTCAGATGCCAGA
	Rev	CCACTTAGCTTTCAGCCACC
Reln (reelin)	Forw	CTCGACAAGCATCCAGTCTTC
	Rev	AGGTTGGTTGTAGGCAGGTG
Sst (somatostatin)	Forw	CCCAGACTCCGTCAGTTTCT
	Rev	CCAGGGCATCATTCTCTGTC
Tfrc (transferrin receptor)	Forw	CCAGTGTGGGAACAGGTCTT
	Rev	GCACCAACAGCTCCAAAGTC
Vip (vasoactive intestinal peptide)	Forw	GGAGCAGTGAGGGAGATTCTG
	Rev	CGTGGTTGTTTTCCTTCGAG

### Western blot analysis of NMDA receptor subunits

GluN1, GluN2A, GluN2B, and GluN2D receptor subtype protein levels were examined in the prefrontal cortex and hippocampus at PND9, PND21 and PND75. Tissue was dissected out and homogenized at 4 °C in a buffered solution and used for Western blot analysis as reported previously [35]. The following primary antibodies were used: rabbit monoclonal anti-NMDAR1 (Abcam, Cambridge, UK #ab109182; 1:5000); rabbit monoclonal anti-NMDAR2A (Abcam, #ab14596; 1:1500); mouse monoclonal anti-NMDAR2B (Abcam, #ab28373; 1:500); rabbit monoclonal anti-NMDAR2D (Abcam, #ab35448; 1:1000); mouse monoclonal anti-GAPDH (Santa Cruz Biotechnology, Dallas, Texas, sc-32233; 1:1000).

Immunostaining was revealed by the enhanced ECL Western blotting analysis system (Hybond ECL, GE Healthcare Europe) and by the Chemidoc computerized densitometer (Bio-Rad). The Immunoblots signal was quantified by ImageJ software.

### Assessment of MK-801-stimulated locomotor activity

(5S,10R)-(+)-5-Methyl-10,11-dihydro-5H-dibenzo[a,d]cyclohepten-5,10-imine maleate (MK-801), was purchased from Tocris Bioscience (Bristol, U.K.). MK-801-induced hyperactivity was assessed in an open-field apparatus. The latter consisted of an unfamiliar cubic box (42 × 42 × 21 cm) with its top left uncovered and transparent plastic walls. The box was connected to an Activity Monitor equipped with infrared photobeam interruption sensor and animal movements were measured and recorded by a computerized analysis system (Open Field Activity System Hardware; Med Associates, Inc., St. Albans, U.K.). On the day of the experiment mice were transferred to the testing room and left in the open field apparatus to record basal locomotor activity for 60 min. Afterwards, they were treated i.p. with MK-801 (0.32 mg/kg or 0.64 mg/kg) and positioned back into the box, where locomotor activity was recorder for additional 120 min defined as “hyperactivity phase”. The software was set to record the distance travelled by mice every 5 min for a total of 180 min. Locomotor activity was expressed as a function of time in 5 min beans (line graphs) or accumulated activity during the habituation phase and hyperactivity phase.

### Statistical analysis

Statistical analysis of biochemical data was carried out by Student’s t test. Statistical analysis for locomotor activity was carried out by One-way ANOVA for repeated measures followed by Fisher’s Least Significant Difference (LSD).

## Results

### ***Developmental profile of interneuron-related genes in the prefrontal cortex and hippocampus of mGlu5***^***−/−***^*** mice***

We first measured the transcripts of interneuron-related genes in the prefrontal cortex and hippocampus of mGlu5^−/−^ mice and their wild-type littermates at three developmental stages (PND9, PND21, and PND75). We focused on GAD1 and GAD2, encoding the two isoforms of the GABA-synthesizing enzyme, glutamate decarboxylase (GAD67 and GAD65) respectively; Pvalb and SSt, encoding PV and somatostatin (SSt), which are expressed by the two major interneuron populations originating from the medial ganglionic eminence; and Reln and Vip, encoding Reelin and vasoactive intestinal peptide (Vip), as representative biomarkers of interneurons originating from the caudal ganglionic eminence [36].

**Fig. 1:**
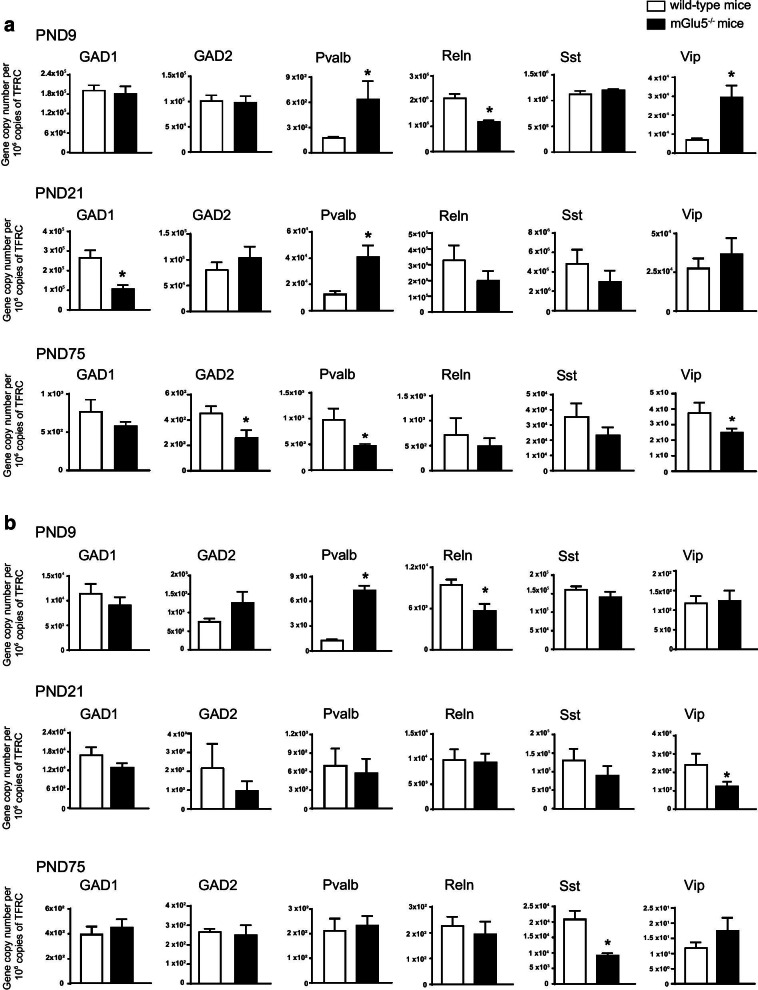
Influence of genetic deletion of mGlu5 receptors on the expression of interneuron-related genes in the prefrontal cortex and hippocampus across postnatal development. mRNA levels encoding interneuron-related proteins in the prefrontal cortex and hippocampus at PND9 -21 and -75 are shown in **a** and **b**, respectively. Values are means ± S.E.M. of 5–9 mice per group. * p< 0.05 vs. the corresponding values of wild-type littermates (Student’s t-test). **a** PND9: Pvalb, t_10_ = 2.3; Reln, t_10_ = 5.6; Vip t_10_ = 5.6; PND21: GAD1, t_11_ = 4; Pvalb, t_11_ = 3.1; PND75: GAD2, t_5_ = 2.8, Pvalb, t_5_ = 3.2, Vip, t_5_ = 2.6. **b** PND9: Pvalb, t_8_ = 11.1; Reln, t_7_ = 3.5; PND21: Vip, t_9_ = 2.2; PND75: SSt, t_6_ = 6.2

- *Prefrontal cortex*
  Changes in Pvalb and Vip expression in mGlu5^−/−^ mice were not uniform in the three selected developmental time points. Pvalb expression was significantly increased at PND9 and PND21, and reduced at PND75, as compared to wild-type littermates (Fig. 1a). Similarly, Vip expression showed a significant increase at PND9, a trend to an increase at PND21, and a significant reduction at PND75 (Fig. 1a). Reln expression in mGlu5^−/−^ mice was exclusively reduced at PND9, whereas SSt expression did not differ between the two genotypes at all developmental time points (Fig. 1a). GAD1 was reduced in mGlu5^−/−^ mice at PND21, whereas GAD2 was reduced at PND75 (Fig. 1a).
- *Hippocampus*
  Changes in the expression of interneuron-related genes were less substantial in the hippocampus of mGlu5^−/−^ mice compared to wild-type littermates across postnatal development. Similarly to the prefrontal cortex, Pvalb expression was significantly increased in mGlu5^−/−^ mice at PND9, but did not change at PND21 and PND75 (Fig. 1b). Reln expression was reduced in mGlu5^−/−^ mice at PND9, whereas Vip and SSt expression was reduced at PND21 and PND75, respectively (Fig. 1b). No other differences in gene expression between mGlu5^−/−^ mice and wild-type littermates were found in the hippocampus (Fig. 1b).

### ***Developmental change in the expression of NMDA receptor subunits in mGlu5***^***−/−***^*** mice***

We studied the expression of NMDA receptor subunits in the prefrontal cortex and hippocampus of mGlu5^−/−^ mice and wild-type littermates because NMDA receptors are structurally and functionally coupled to mGlu5 receptors, and are highly functional in GABAergic interneurons (see “Introduction” and References therein). We found substantial changes in the transcript and protein levels of NMDA receptor subunits in both brain regions of mGlu5^−/−^ mice at PND9 and PND21 but not in the adult life (PND75).

**Fig. 2:**
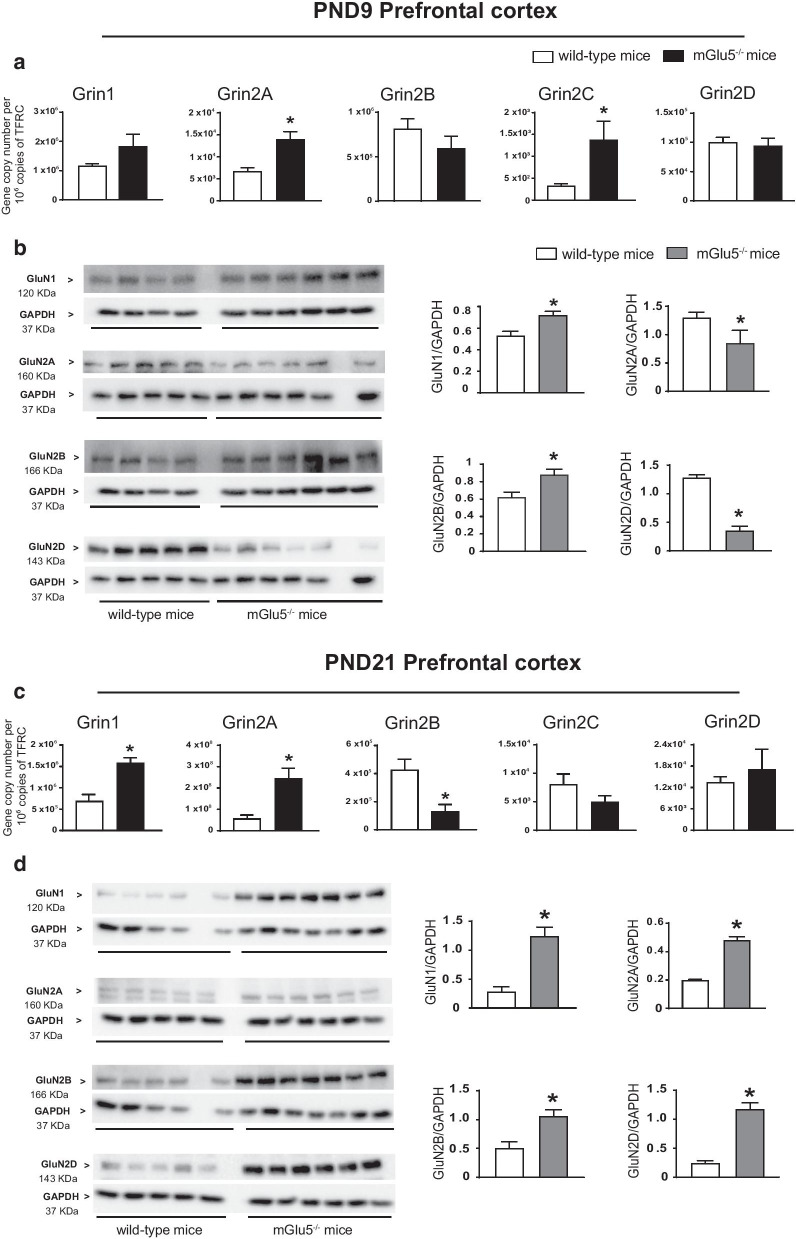
Expression of NMDA receptor subunits in the prefrontal cortex of mGlu5^−/−^ mice and wild-type littermates at PND9 and PND21. mRNA and protein levels at PND9 and PND21 are shown in **a**, **c** and **b**, **d**, respectively. Values are means ± S.E.M. of 4–9 mice per group. *p < 0.05 (Student’s t-test). **a** Grin2A, t_10_ = 3.8; Grin2C, t_9_ = 2.8; **b** GluN1, t_8_ = 3.4; GluN2A, t_9_ = 9.4; GluN2B, t_8_ = 2.8; GluN2D, t_9_ = 9.4 **c** Grin1, t_12_ = 4.6; Grin2A, t_13_ = 2.5; Grin2B, t_12_ = 3.4 **d** GluN1, t _9_ = 7; GluN2A, t_8_ = 4.1; GluN2B, t_10_ = 3.4; GluN2D, t_9_ = 7. Uncropped western blots are shown

**Fig. 3:**
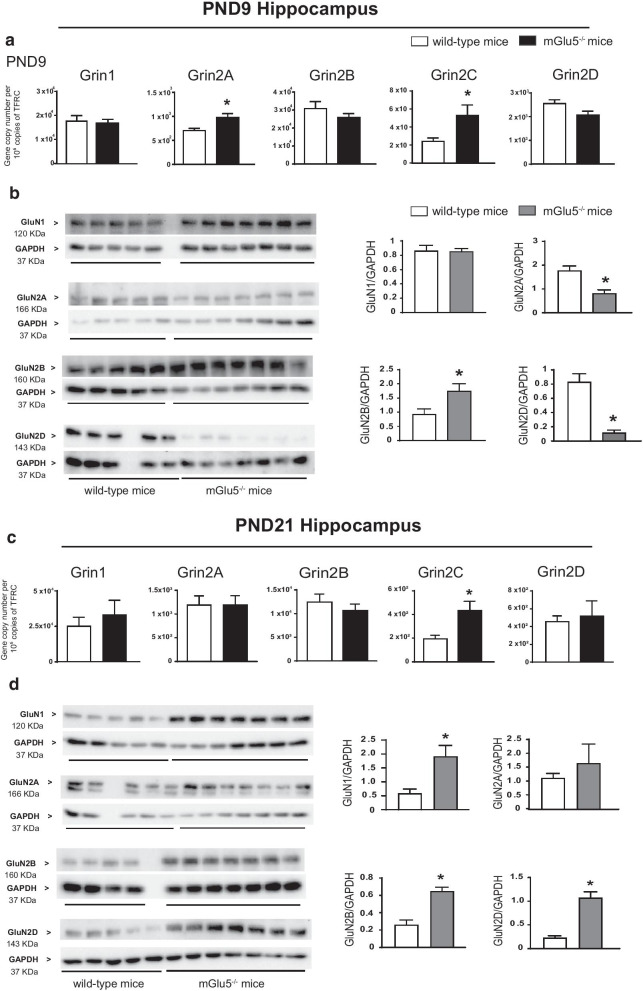
Expression of NMDA receptor subunits in the hippocampus of mGlu5^−/−^ mice and wild-type littermates at PND9 and PND21. mRNA and protein levels at PND9 and PND21 are shown in **a**–**d**. Values are means ± S.E.M. of 4–9 mice per group. *p < 0.05 (Student’s t-test). **a** Grin2A, t_9_ = 3.5; Grin2C, t _9_ = 2.5; **b** GluN2A, t_10_ = 4; GluN2B, t_9_ = 2.7; GluN2D, t_10_ = 7; **c** Grin2C, t_11_ = 2.5; **d** GluN1, t_10_ = 2.8; GluN2B, t_9_ = 5.2; GluN2D, t_10_ = 5.2. Uncropped western blots are shown

**Fig. 4:**
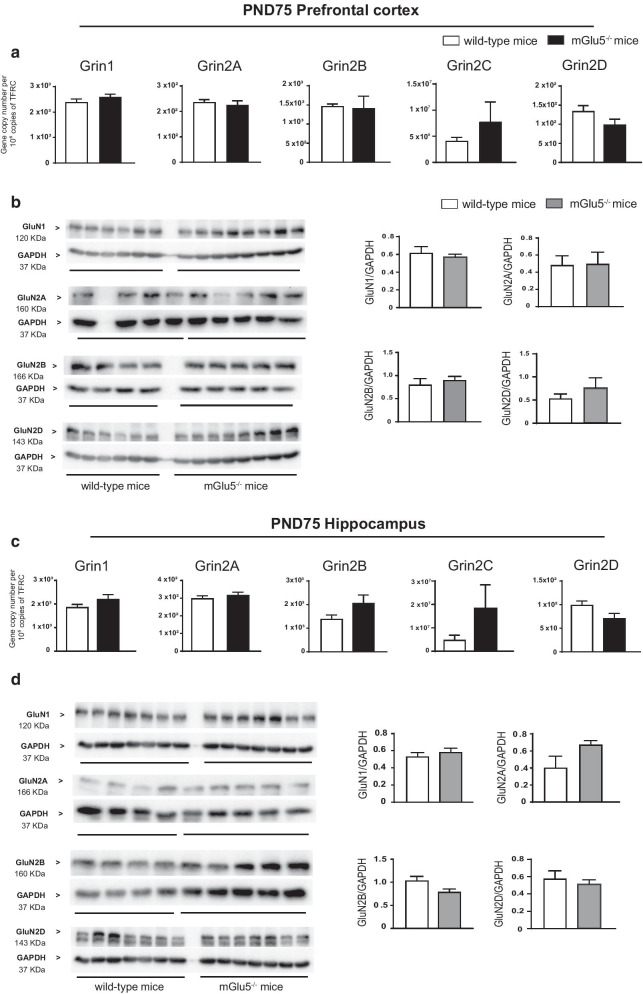
Expression of NMDA receptor subunits in the prefrontal cortex and hippocampus of mGlu5^−/−^ mice and wild-type littermates at PND75. mRNA and protein levels in the prefrontal cortex and hippocampus are shown in **a**–**d**. Values are means ± S.E.M. of 4–6 mice per group. Uncropped western blots are shown

- *Prefrontal cortex *
  At PND9, there was only a partial correspondence between transcript and protein levels of the five selected NMDA receptor subunits (GluN1, and Gln2A-D). Expression of Grin2A and Grin2C genes, encoding GluN2A and GluN2C subunits, respectively, was largely increased in mGlu5^−/−^ mice, as compared to wild-type littermates, whereas the other transcripts did not change (Fig. 2a). Immunoblot analysis showed a significant increase in GluN1 and GluN2B protein levels, whereas GluN2A and GluN2D levels were significantly reduced in mGlu5^−/−^ mice (Fig. 2b). We were unable to detect the GluN2C subunit under our conditions (using three commercially available antibodies).
  It was at PND21 that we found the largest changes in NMDA receptor subunits in mGlu5^−/−^ mice. At this time point, expression of Grin1 and Grin2A was largely increased, whereas Grin2B expression was nearly suppressed (Fig. 2c). Remarkably, mGlu5^−/−^ mice showed a substantial increase in GluN1, GluN2B, and GluN2D protein levels, and a smaller increase in GluN2A levels at PND21 (Fig. 2d). Again, all changes in the transcript and protein levels of NMDA receptor subunits between mGlu5^−/−^ mice and wild-type littermates disappeared at PND75 in the prefrontal cortex (Fig. 4a, b).
- *Hippocampus*
  In the hippocampus, transcript and protein levels of NMDA receptor subunits were divergent at PND9 and PND21. At PND9, Grin2A and Grin2C expression was significantly increased in mGlu5^−/−^ mice, as compared to wild-type littermates (Fig. 3a). In contrast, GluN2B protein levels were increased, GluN2A and GluN2D levels were reduced, and GluN1 levels did not change in mGlu5^−/−^ mice at this time point (Fig. 3b). At PND21 only Grin2C transcript levels showed a significant increase in mGlu5^−/−^ mice (Fig. 3c), whereas protein levels of GluN1, GluN2B and GluN2D were largely increased (Fig. 3d). At PND75, no changes in transcript and protein levels of NMDA receptor subunits were found in mGlu5^−/−^ mice as compared to wild-type littermates (Fig. 4c, d).

### ***Changes in MK-801-induced hyperactivity in mGlu5***^***−/−***^*** mice at PND21 that is inverted at PND75***

**Fig. 5:**
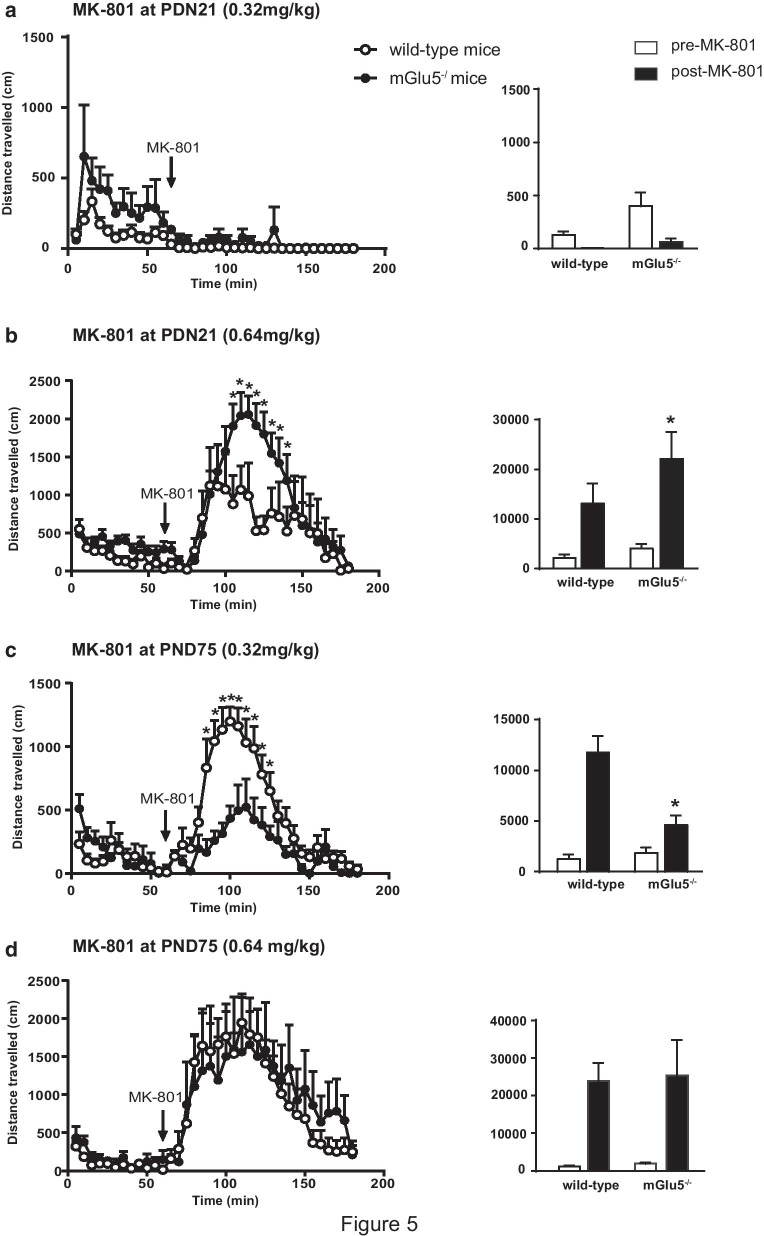
MK-801-induced hyperactivity in mGlu5^−/−^ mice and wild-type littermates at PND21 and PND75. Locomotor activity in response to 0.32 mg/kg or 0.64 mg/kg MK-801 at PND21 and in response to 0.32 mg/kg or 0.64 mg/kg MK-801 at PND75 is shown in **a**–**d**. Mice were habituated to the environment for 60 min prior to the i.p. injection of MK-801. Values are means ± S.E.M. of 10 wild-type and 3 mGlu5^−/−^ mice in **a**, 5 wild-type and 6 mGlu5^−/−^ mice in **b**, 7 wild-type and 5 mGlu5^−/−^ mice in **c**, 7 wild-type and 4 mGlu5^−/−^ mice in **d**. Open bars = habituation phase; closed bars = response to MK-801. *p < 0.05 vs. wild-type mice (One-way ANOVA for repeated measures). **b**, F_1,119_ = 27.875; p < 0.001; **c** F_1,119_ = 44.692; p < 0.001

Moving from the overexpression of NMDA receptor subunits found in mGlu5^−/−^ mice at PND21, we examined whether this could be paralleled by changes in the behavioral response to the slow NMDA channel blocker, MK-801. Of note, MK-801-induced hyperactivity is widely used as an experimental animal model of psychosis [37]. After 60 min of habituation to the open field apparatus, mice were injected with MK-801, and motor activity was recorded for additional 120 min. At PND21, mice did not respond to the standard dose of MK-801 used in our laboratories (0.32 mg/kg, i.p.) (Fig. 5a), and, therefore, we decided to repeat the experiment doubling the dose of MK-801. In response to 0.64 mg/kg of MK-801 both mGlu5^−/−^ mice and wild-type littermates showed a significant increase in locomotor activity, which lasted for at least 90 min. However, the response to MK-801 was much greater in mGlu5^−/−^ mice, with no significant difference between the two genotypes in the habituation phase (Fig. 5b). As expected, adult mice (PND75) responded to 0.32 mg/kg of MK-801, but the increase in locomotor activity was less pronounced in mGlu5^−/−^ mice, as compared to wild-type littermates (Fig. 5c). There was no difference between the two genotypes when PND75 mice were challenged with 0.64 mg/kg of MK-801 (Fig. 5d).

## Discussion

Functional NMDA receptors are heterotetramers typically formed by two GluN1 subunits and two GluN2 subunits. GluN2A, GluN2B; GluN2C, and GluN2D subunits are encoded by four different genes (Grin2A-D) [38, 39]. The GluN1 subunit is ubiquitously expressed with no major developmental changes, whereas the expression pattern of GluN2 subunits shows remarkable changes across postnatal development. The GluN2A subunit starts to be expressed early after birth and expression progressively increases afterwards, whereas the GluN2D subunit shows an opposite expression profile. Expression of the GluN2B subunit is ubiquitous in the first two weeks of postnatal life, and becomes restricted to the forebrain in the adult life. The GluN2C subunit begins to be expressed at PND10, and expression is mainly confined to the cerebellum. The most remarkable developmental change in NMDA receptor composition is the switch between GluN2B- to GluN2A-containing heterotetramers at a time that coincides with synaptic maturation, and circuit refinement [39].

Absolute levels of Grin1 and Grin2A-D transcripts in the prefrontal cortex and hippocampus of wild-type mice changed during postnatal development as reported previously [39], although we were surprised to find high expression levels of Grin2C mRNA at PND75. Expression of NMDA subunit protein levels was only studied comparatively between mGlu5^−/−^ and wild-type littermates without comparing levels of the same strain at the different time points. Interestingly, we found a large overexpression of the GluN2B subunit at both PND9 and PND21 in both prefrontal cortex and hippocampus of mGlu5^−/−^ mice, as compared to wild-type littermates. This was associated with a significant reduction of the GluN2A subunit at PND9, but not at PND21. These findings suggest that the lack of mGlu5 receptors delays the developmental shift between the GluN2B and the GluN2A subunit, which normally occurs after the first 7–10 days of postnatal life [40]. This is in nice agreement with electrophysiological data showing that the developmental switch between GluN2B and GluN2A (formerly named NR2B and NR2A) is defective or absent in the hippocampus and visual cortex of mGlu5 receptor knockout mice [41]. A leading hypothesis is that endogenous activation of mGlu5 receptors promotes the removal of GluN2B-containing NMDA receptors from the plasma membrane through a mechanism mediated by casein kinase 2 [39]. The lack of changes in Grin2B transcript levels between mGlu5^−/−^ and wild-type mice at PND9 supports the hypothesis that mGlu5 receptors regulates the GluN2B/GluN2A balance at protein level [39, 41]. Our data also raise the interesting possibility that mGlu5 receptors critically regulate the expression of the GluN2D subunit, which was largely reduced at PND9 and increased at PND21 in mGlu5^−/−^ mice, with respect to wild-type littermates. The Grin2D transcripts did not change in both regions in the two time points, suggesting again that mGlu5 receptors act at post-transcriptional levels in regulating Grin2D mRNA translation and/or GluN2B protein stability. This hypothesis warrants further investigation.

We could only measure the transcript of Grin2C, which was largely increased in the prefrontal cortex at PND9 and in the hippocampus at both PND9 and PND21. The significance of these changes is uncertain because, at least in the hippocampus and cerebral cortex, GluN2C is not expressed by neurons but co-localizes with astrocytic markers [42].

At least in the prefrontal cortex, the general overexpression of NMDA receptor subunits might contribute to explain the unexpected increase in Pvalb transcript observed at PND9 and PND21. In fast-spiking PV^+^ interneurons NMDA receptors are constitutively active owing to membrane depolarization, which removes the Mg^2+^ blockade of the NMDA-gated ion channel (see “Introduction” and References therein). An increased expression of NMDA receptors in the prefrontal cortex of mGlu5^−/−^ mice at PND9 and PND21 might cause a hyperactivation of PV^+^ interneurons with a resulting increase in Pvalb gene expression [43]. This might represent a compensatory mechanism aimed at buffering the increased influx of extracellular Ca^2+^ through the NMDA-gated ion channel. In the hippocampus, the increase in the transcript encoding PV was found at PND9, when an increase in GluN2B subunit was associated with a reduction in GluN2A and GluN2D, and no changes in GluN1 protein levels. In contrast, no changes in Pvalb transcript were seen at PND21, when GluN1, GluN2B, and GluN2D (but not GluN2A) protein levels were increased. It is possible that the presence of the GluN2A subunit is required for the regulation of Pvalb expression by NMDA receptors at PND21 (but not at PND9). This is supported by data obtained in cultured cortical neurons, in which selective blockade of GluN2A-containing NMDA receptors decreased PV and GAD67 immunoreactivity [43].

The transcripts encoding Vip and Reelin, which are two makers of interneurons originating from the caudal portion of ganglionic eminences [36], showed changes at PND9, with Reln transcript being  decreased in both prefrontal cortex and hippocampus, and Vip transcript increased in the prefrontal cortex. At PND75, there was a reduction in the transcript encoding PV, GAD65, and VIP in the prefrontal cortex, and a reduction in the transcript encoding SSt in the hippocampus. These results are partially consistent with a previous report [18] showing a reduction in PV mRNA levels in the prefrontal/frontal cortex (but not in the hippocampus), a reduction in SSt mRNA levels in the hippocampus, and a reduction in GAD65 and GAD67 mRNA levels in the prefrontal cortex/frontal cortex and hippocampus of adult (PND > 80) male mGlu5^−/−^ mice. In our study, protein levels of NMDA receptor subunits were normalized in mGlu5^−/−^ mice at PND75, and this might have disclosed an intrinsic defect in PV expression in the prefrontal cortex and SSt expression in the hippocampus caused by the lack of mGlu5 receptors. Changes in the Pvalb and SSt transcripts seen in adult mGlu5^−/−^ mice are consistent with the view that these mice recapitulate some of the biochemical and behavioral hallmarks of schizophrenia [18, 19, 34].

The most relevant finding of our study was that mGlu5^−/−^ mice showed an impressive increase in GluN1, GluN2A, GluN2B, and GluN2D protein levels in the prefrontal cortex and hippocampus at the time of weaning (PND21), while levels returned back to normal in the adult life. This indicates that the lack of mGlu5 receptors is compensated by an increased expression of its receptor partner (the NMDA receptor) during postnatal development, to limit the potential abnormalities in network activity resulting from the absence of mGlu5 receptors in interneurons. However, this compensatory mechanism is transient and not sufficient to avoid the development of a pathological phenotype in the adult life. We reasoned that, if overexpression of NMDA receptors was a compensatory mechanism at PND21, we could unmask a psychotic-like behavioral phenotype by blocking NMDA receptors. For this reason, we assessed locomotor activity in response to the slow NMDA channel blocker, MK-801. MK-801-induced hyperactivity in rodents is widely used as a model for positive symptoms of schizophrenia [44], which is sensitive to both classical and atypical antipsychotic drugs [45]. Under our experimental conditions, wild-type mice at PND21 did not respond to conventional doses of MK-801 (0.32 mg/kg) [46], and, therefore, a double dose was required for the induction of hyperactivity in these mice. Interestingly, mGlu5^−/−^ mice at PND21 displayed a large increase in MK-801-induced hyperactivity as compared to wild-type littermates, supporting the hypothesis that the overexpression of NMDA receptors represents an important compensatory mechanism in mGlu5^−/−^ mice at this developmental stage. The situation was reversed in the adult life, when hyperactivity was largely attenuated in mGlu5^−/−^ mice in response to 0.32 mg/kg MK-801. A possible explanation is that, in adult mGlu5^−/−^ mice, normally expressed NMDA receptors are less active in supporting the function of fast-spiking, PV^+^, interneurons because of the lack of the mGlu5 receptor partner. As a consequence, the behavioral response to NMDA receptor blockade was blunted in these mice.

## Conclusions

In conclusion, our findings further demonstrate a close partnership between mGlu5 and NMDA receptors in the mouse prefrontal cortex and hippocampus, showing that the lack of mGlu5 receptors was compensated by a large but transient overexpression of NMDA receptor subunits during postnatal development. This may have important implications in the behavioral, biochemical, and electrophysiological phenotype of mGlu5^−/−^ mice, and should be taken into account when these mice are used for the study of mechanisms of developmental plasticity.

## Data Availability

The data that support the findings of this study are available from the corresponding author upon reasonable request.

## Abbreviations

NMDA: N-methyl-d-aspartate receptor
mGlu5 receptor: Metabotropic glutamate receptor type 5
PND: Postnatal day
GluN1,2A-D: N-methyl-d-aspartate receptor subunit 1,2A-D
MK-801: (5S,10R)-(+)-5-Methyl-10,11-dihydro-5H-dibenzo[a,d]cyclohepten-5,10-imine maleate
PI: Polyphosphoinositide hydrolysis
CNS: Central nervous system
GABA: Gamma-aminobutyric acid
PV: Parvalbumin
PNN: Perineuronal nets
TFRC: Transferrin receptor
GAPDH: Glyceraldehyde 3-phosphate dehydrogenase
ECL: Enhanced chemiluminescence
LSD: Least significant difference
GAD1: Glutamate decarboxylase 1
GAD2: Glutamate decarboxylase 2
Pvalb: Parvalbumin gene
SSt: Somatostatin gene
Reln: Reelin gene
Vip: Vasoactive intestinal peptide gene
Grin1,2A-D: N-methyl-d-aspartate receptor subunit 1, 2A-D gene
Mg^2^^+^: Magnesium
Ca^2^^+^: Calcium
