# Correction to: Developmental up-regulation of NMDA receptors in the prefrontal cortex and hippocampus of mGlu5 receptor knock-out mice

**DOI:** 10.1186/s13041-021-00822-6

**Published:** 2021-07-16

**Authors:** Tiziana Imbriglio, Remy Verhaeghe, Nico Antenucci, Stefania Maccari, Giuseppe Battaglia, Ferdinando Nicoletti, Milena Cannella

**Affiliations:** 1 IRCCS Neuromed, Pozzilli, IS Italy; 2 Department of Physiology and Pharmacology “V. Erspamer”, University Sapienza of Rome, Piazzale Aldo Moro, 5, 00185 Rome, Italy; 3 CNRSUMR 8576, UGSFUnité de Glycobiologie Structurale et Fonctionnelle, University of Lille, Lille, France

## Correction to: Mol Brain (2021) 14:77 https://doi.org/10.1186/s13041-021–00784-9

Following publication of the original article [1], the authors identified an error in the affiliation assignment for the third author, Nico Antenucci. This author was incorrectly assigned to affiliation 1 (IRCCS Neuromed, Pozzilli, IS, Italy) instead of affiliation 2 (Department of Physiology and Pharmacology “V. Erspamer”, University Sapienza of Rome, Piazzale Aldo Moro, 5, 00,185 Rome, Italy).

The affiliation list has been updated above and the original article [1] has been corrected.
